# Jubilee of the Hospital Saturday Fund

**Published:** 1923-05

**Authors:** 


### JUBILEE OF THE HOSPITAL SATURDAY FUND.

### RAISING A MILLION FOR HOSPITALS.

THE annual meeting of the Hospital Saturday Fund, which was held at the Mansion House, on April 14, was of peculiar interest, since this is the jubilee year of the Fund. A letter was received from the King's Secretary, Lord Stamfordham, expressing the hope that success would attend the special efforts that are being made to commemorate the fiftieth year of the Fund's activities. The Lord Mayor, who presided, said that during the Fund's existence over £1,000,000 had been collected and distributed among hospitals. The Earl of Malmesbury moved the adoption of the report, which showed that £75,775 was distributed amongst 222 hospitals and allied institutions during 1922, compared with £81,000 in 1921. In view of the industrial depression existing during the year, the results achieved were most satisfactory.

### The Awards.

The following is a summary of the awards that have been made :—

| | | |
|---|---|---|
| 45 | general hospitals .. .. .. | £27,051 |
| 23 | cottage hospitals .. .. .. | 2,700 |
| 77 | special hospitals .. .. .. | 23,699 |
| 19 | dispensaries .. .. .. .. | 319 |
| 20 | convalescent homes .. .. .. | 5,179 |
| 27 | nursing institutions .. .. .. | 893 |
| 11 | miscellaneous (including dental, distribution, surgical appliance, and Ambulance committees) .. .. | 15,934 |
| 222 | | £75,775 |

The amount awarded in 1921 was £81,005.

### The Year's Work.

During the past year there were 4,452 applications for dental assistance, the cost being £16,809, towards which £12,410 was received; 1,837 convalescents were sent to suitable homes, 1,802 of whom paid about 10s. a week towards the cost of their maintenance. In addition numerous grants were made to patients who were sent to convalescent homes through hospitals or similar institutions. The Surgical Appliance Committee assisted in the provision of 14,148 appliances, including spectacles, at a cost of £9,509, towards which the recipients contributed £5,828. Grants were made towards the cost of 1,015 appliances supplied through hospitals, and of 4,073 pairs of spectacles supplied through opticians in the outlying suburbs. The number of benefits granted during the year was 67,509, as against 62,378 in the preceding year. The management expenses of the fund amounted to £8,333, or 8·6 per cent. of the gross receipts.

### Story of the Fund.

The London Hospital Saturday Fund was founded in the autumn of 1873, following closely the Metropolitan Hospital Sunday Fund, which was founded in the spring of the same year. Some ardent men, imbued with the sense of brotherhood, realising that the older fund failed to obtain help from many who did not attend a place of worship on Hospital Sunday, and that there were other ways of helping the industrial classes in matters relating to health, decided to form a separate fund. Under the leadership of such men as Lord Brabazon, Captain Charles Mercier and Mr. H. N. Hamilton Hoare the new association was established and gradually worked its way into the hospital life of the Metropolis.

### Sources of Income.

The Fund has always had two main sources of income—first, that obtained by means of weekly and other systematic collections in the workshops and business houses of London ; and, secondly, the annual collections of Hospital Saturdays. In the first fifteen years of its existence the annual income of the Fund increased slowly, but a successful effort was made by Sir James Whitehead, then Lord Mayor, to develop the penny-a-week collection in workshops and business houses. As this collection increased the annual street collection declined, and this, on the advice of Sir R. B. D. Acland, was finally discontinued. The income then progressed steadily for several years, until the advent of the National Insurance Act gave it a temporory set-back.

### How the Fund is Managed.

The Fund is managed by a Board of Delegates elected by subscribers in the workshops and business houses, and by the Local Committees, with the additions of a representative from each institution receiving a grant. These delegates in turn elect five Committees, called the Ambulance, Collection, Distribution, Finance and Surgical Appliance Committees; each Committee has an Honorary Secretary, and these Honorary Secretaries, together with the Chairman of the Fund, the Chairman of the Council, Treasurer, and a representative of each Committee form the Executive Committee. The Board of Delegates meets quarterly to receive the reports of the Committees and to decide principles, and two or three times a year in addition to transact special business. The Committees meet weekly, fortnightly or monthly as their duties require. The principle underlying the whole of the work done, independently of the hospitals, has been "self-help." Beds have been endowed, convalescents sent to homes, dentists appointed, surgical appliances supplied. Classes in First Aid, Nursing and Hygiene are arranged, and Ambulance and Nursing divisions of the St. John's Ambulance Brigade were formed, which were associated with and assisted by the Fund. During its fifty years of life the Fund has done its best to promote the national welfare by supporting the hospitals and kindred institutions, and by supplementing their work. It has in the past helped to solve difficult problems connected with hospital affairs, and its work has been the expression of the desire of many among the working classes to take their proper share in the maintenance of our great hospitals.

